# Atmospheric Corrosion Analysis and Rust Evolution Research of Q235 Carbon Steel at Different Exposure Stages in Chengdu Atmospheric Environment of China

**DOI:** 10.1155/2020/9591516

**Published:** 2020-02-19

**Authors:** Zhigao Wang, Mei Wang, Jie Jiang, Xinsheng Lan, Fangqiang Wang, Zhi Geng, Qianqian Tian

**Affiliations:** ^1^State Grid Sichuan Electric Power Research Institute, Chengdu 610041, China; ^2^School of Mechanical Engineering, Sichuan University, Chengdu 610065, China; ^3^State Key Laboratory of Geohazard Prevention and Geoenvironment Protection (Chengdu University of Technology), Chengdu 610059, China; ^4^College of Environment and Civil Engineering, Chengdu University of Technology, Chengdu 610059, China

## Abstract

In order to effectively reduce and retard corrosion of the power transmission and transformation equipment in Chengdu power grid and to improve power supply reliability, Q235 carbon steel material which is the most widely used metal material in power grid was selected as the targeted research object in this article. Exposure experiments were performed in urban atmospheric environment of Chengdu city in the southwest region of China. The corrosion behavior of Q235 carbon steel material was investigated at different seasons. The macro- and micromorphologies after corrosion were observed using a digital camera and scanning electron microscopy (SEM), respectively. Element distribution of the rust layer and the corrosion products was characterized by energy-dispersive spectroscopy (EDS), X-ray diffraction (XRD), and Fourier transform infrared spectroscopy (FTIR); the corrosion mechanism was also briefly analyzed.

## 1. Introduction

In recent decades, with the construction scale of China's power transmission and transformation project becoming larger and larger, the corrosion problem of power transmission and transformation equipment is increasingly prominent, which has seriously affected safe operation of the power grid system [1–7]. With long-term operation outdoor, the metal components of the power transmission and transformation equipment are subject to erosion and damage due to various harsh environments and are prone to corrosion and failure, reducing the reliability of the power transmission and transformation equipment and generating potential safety hazards [8–10]. For instance, the transformer radiator in a 500 kV substation suffered corrosion perforation, resulting in oil leakage malfunction, which had to be replaced after power failure. In another 220 kV substation, corrosion of the outdoor terminal cabinet caused bad sealing and moisture in the cabinet, which easily caused component fault and equipment misoperation. Corrosion of the high-voltage disconnecting switch at the connecting rod and gear plate parts in a 110 kV substation caused the operating mechanism to be jammed, and the switch could not reach the designated position. In a 220 kV transmission line, corrosion failure of the electric power fitting led to the conductor disconnecting or falling, causing power outage accidents.

Chengdu is a megacity in the southwest region of China with a population of 16.04 million and an area of 14,600 square kilometers. For Chengdu power grid, there are 410 substations above 35 kV with a total substation capacity of 61.76 million kVA, and there are 8808 km of transmission lines above 35 kV. Chengdu has a humid and rainy climate with the annual average temperature of 16.6°C and the annual average relative humidity of 81%; also, its annual average rainfall is 966.9 mm [11]. In addition, the air environment in Chengdu has been seriously polluted in recent years; the major pollutants include SO_2_, NO_2_, O_3_, CO, PM_10_, and PM_2.5_ [12]. Both the climatic and environmental factors will accelerate the corrosion rate of the metal materials. The interaction of various factors influencing corrosion is complex, reflecting dynamic and evolutive characteristics for the corrosion of the metal materials. The diversity of the atmospheric pollutants also leads to difference in the corrosion layer. However, corrosion studies concerning the metal materials used in power grid in the specific atmospheric environment of Chengdu city are very scarce. Thus, it is essential to investigate the corrosion behavior of the metal materials in Chengdu atmospheric environment, which is very important to guarantee safe operation of the power transmission and transformation equipment in Chengdu power grid.

In this study, on the basis of rainy, high-humidity, and high-acidic pollution characteristics of the atmospheric corrosion environment in Chengdu, the corrosion behavior and regularity of Q235 carbon steel material which has been widely used in power transmission and transformation projects were evaluated after long-term exposure to ambient air. In particular, formation and evolution mechanism of the corrosion layer of Q235 carbon steel at different exposure stages were researched. The findings obtained in this study will be helpful to develop targeted corrosion protection strategy for urban atmospheric environment in Chengdu, for the purpose of effectively controlling corrosion and ensuring safe and stable operation of Chengdu power grid.

## 2. Materials and Methods

### 2.1. Materials and Sample Preparation

Q235 carbon steel material was selected for corrosion tests in this study. Q235 carbon steel material was one of the most widely used metallic materials in power transmission and transformation projects. The chemical composition of Q235 carbon steel is shown in Table 1.

All the Q235 carbon steel samples used in atmospheric exposure experiments were cut to 100 mm × 50 mm × 3 mm by wire electrode cutting. Then, they were ground by machinery grinding to 800-grit smooth surface. The surface oil was cleaned by an ultrasonic cleaner in anhydrous alcohol, and then, the specimens were dried with a blow dryer and stored in a drying vessel.

### 2.2. Exposure Experiments

The atmospheric exposure experiments were undertaken at Chengdu atmospheric corrosion station of China. The prepared Q235 carbon steel specimens were installed on a test rack with an inclination angle of 45° and exposed to ambient air, horizontal to the sky and facing south, where they were exposed for one year (from Nov 2016 to Nov 2017). The environmental parameters such as temperature, relative humidity, rainfall precipitation, pH value of rainfall, SO_2_ concentration, and Cl^−^ settling rate during the atmospheric exposure tests in Chengdu are listed in Table 2.

Q235 carbon steel specimens were collected each time after exposure to ambient air for consecutive 15, 30, 90, 180, and 365 days, respectively. They were used to analyze the corrosion process, corrosion morphology, and rust layer products.

### 2.3. Macroscopic Corrosion Morphology Observation

Using a digital camera (Canon, PowerShot SX700 HS) to take macroscopic pictures of the Q235 carbon steel specimens after 15, 30, 90, 180, and 365 days of exposure in Chengdu atmospheric environment, the macroscopic corrosion morphologies were observed.

### 2.4. Scanning Electron Microscope and Element Analysis

The microstructures and the cross-section morphologies of the Q235 carbon steel specimens were observed by SEM (Hitachi, SU3500). Energy-dispersive spectroscopy (EDS, Hitachi) was used to determine the elements of the corrosion products.

### 2.5. Corrosion Product Analysis

The crystalline phase of the rust layer was identified using a powder X-ray diffractometer (XRD, Empyrean). The rust layer was removed from the specimen and fully ground in an agate mortar body with 5 *μ*m particle size. The 2 theta angle was 10°~90°. The results of XRD were analyzed by Jade 6.0 software.

### 2.6. FTIR Analysis

The composition of the rust layer was analyzed by Fourier transform infrared spectroscopy (FTIR) on a Nicolet 6700 FTIR spectrophotometer (Nicolet, USA) between 4000 and 500 cm^−1^ with a resolution of 2 cm^−1^.

## 3. Results and Discussion

### 3.1. Macroscopic Corrosion Morphology

**Figure 1:**
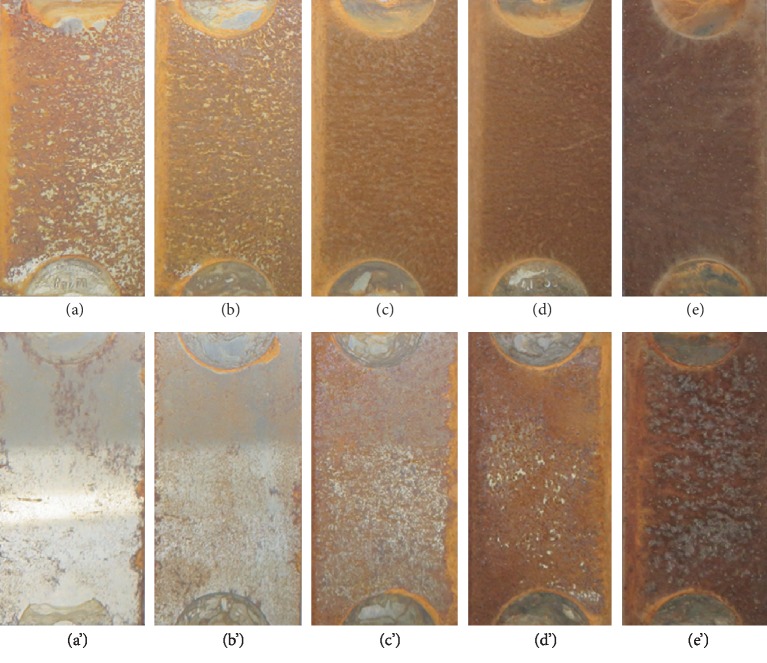
Macroscopic corrosion morphologies of the Q235 carbon steel specimens with different exposure time in Chengdu station: (a) 15 days on the front side, (a′) 15 days on the back side, (b) 30 days on the front side, (b′) 30 days on the back side, (c) 90 days on the front side, (c′) 90 days on the back side, (d) 180 days on the front side, (d′) 180 days on the back side, (e) 365 days on the front side, and (e′) 365 days on the back side.

Q235 carbon steel specimens were exposed in Chengdu atmospheric environment for 15, 30, 90, 180, and 365 days, respectively. The corrosion weight loss after 365 days of exposure was 17.42 *μ*m/a. The retrieval samples were photographed with a digital camera.  Figure 1 shows the macroscopic corrosion morphologies of the Q235 carbon steel samples with different exposure time in Chengdu station. Figures 1(a)–1(e) represent the corrosion morphologies on the front sides of the specimens at different exposure time, while Figures 1(a′)–1(e′) represent the corrosion morphologies on the back sides of the specimens at different exposure time. When the Q235 carbon steel material was exposed for 15 days, most region of the specimen on the front side had been overlaid by a rust layer as shown in Figure 1(a); part of the substrate metal was still bared; the surface appeared to have uneven brown color.  Figure 1(a′) shows the corrosion morphology of the Q235 carbon steel specimen on the back side; the corrosion degree of the back side was far lower than that of the front side; there were few rust spots; most region of the substrate metal was still naked. After exposure for 30 days, Figure 1(b) displays the corrosion morphology on the front side of the specimen; the area of the rust layer continued to expand; it could be found that bared substrate metal decreased significantly. At the same time, on the back side of the specimen shown in Figure 1(b′), the number of the rust spots increased and the region of the rust layer was enlarged; however, the corrosion degree of the back side was still much lower than that of the front side. When the Q235 carbon steel specimen was exposed for 90 days, Figure 1(c) exhibits that the front side of the specimen had been completely covered by the brown rust layer and the outer rust layer was loose.  Figure 1(c′) shows the back-side morphology of the specimen; the number of the rust spots was obviously increasing and intensive, but they had not completely covered the substrate metal. At the exposure time of 180 days, the front side of the specimen presented a homogeneous brown rust layer, and compared with the early exposure periods, the rust layer was becoming denser (Figure 1(d)).  Figure 1(d′) presents that the corrosion degree of the back side of the specimen continued to grow; the rust layer had almost completely covered the substrate metal; only a small amount of substrate metal remained uncovered. When the Q235 carbon steel specimen was exposed for 365 days, Figure 1(e) shows that the front side of the specimen appeared dark brown color and demonstrated a compact rust layer. In Figure 1(e′), the corrosion degree of the back side of the specimen had been significantly increased compared with that of 180 days, the rust layer presented reddish brown color, and its outer rust layer was more loose than that of the front side. Through the morphological observation from the macroscopic angle, in the earlier exposure periods of 15 days~180 days, the corrosion degree of the Q235 carbon steel specimen on the front side was more serious than that on the back side. This is because the corrosion on the front side of the specimen was mainly caused by rain water, condensation, and moist atmosphere. However, on the back side of the specimen, the rain water and condensation were difficult to attach, so its corrosion was principally caused by moist atmosphere. In the later exposure period of 365 days, the front side of the specimen had formed a relatively dense rust layer, moisture was not easily to permeate, so the corrosion growth slowed down. While the back side of the specimen was covered by a relatively loose rust layer, moisture could be easily permeated through the outer rust layer, so the corrosion continued to grow. In addition to exposure time, season also affected the corrosion process. The initial 15 days and 30 days were in winter, with less rainfall and low humidity, and led to low corrosion degree. 90 days of exposure was in spring; the corrosion degree was higher than that in winter. 180~365 days were in summer and autumn, with the most rain and the highest corrosion degree.

### 3.2. Microscopic Corrosion Morphology

**Figure 2:**
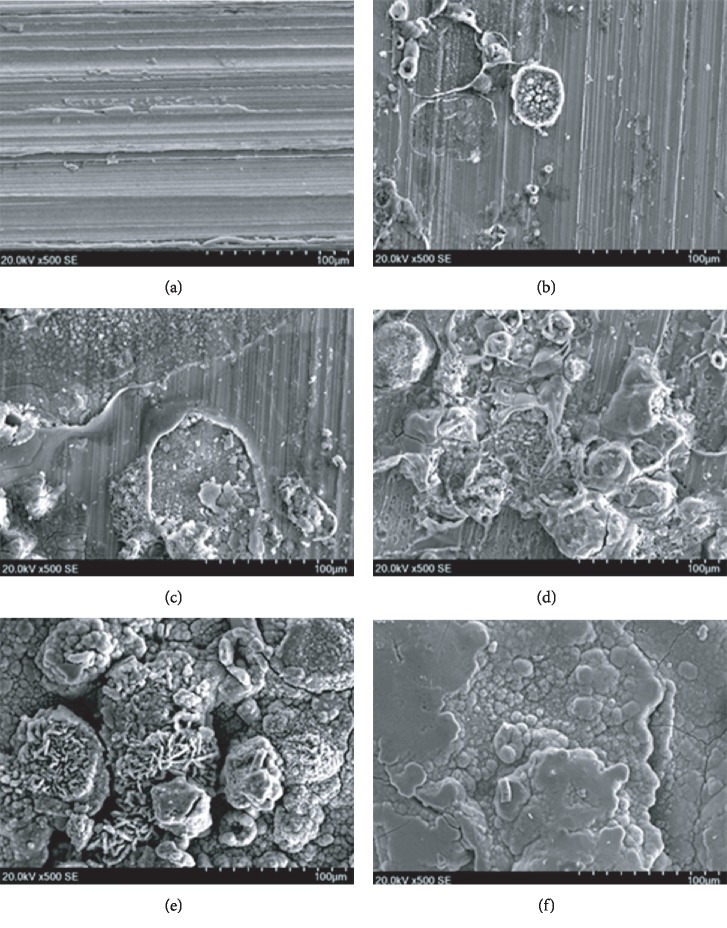
Microscopic corrosion morphologies of the Q235 carbon steel specimens with different exposure time in Chengdu station: (a) 0 day, (b) 15 days, (c) 30 days, (d) 90 days, (e) 180 days, and (f) 365 days.

SEM was performed to observe the surface microscopic corrosion morphologies of the Q235 carbon steel samples with different exposure time in Chengdu station; the results are shown in Figure 2. Figures 2(a)–2(f) represent the front-side surface morphologies of the Q235 carbon steel samples exposed for 15, 30, 90, 180, and 365 days, respectively. The SEM magnifications were all set as 500 times.

It can be observed from Figure 2(a) that the Q235 carbon steel specimen before exposure showed a striated surface after magnification, which was due to the surface polishing to the substrate metal. When the Q235 carbon steel sample was exposed for 15 days, several circular areas in Figure 2(b) were local corrosion points, where the corrosion products were composed of spherical particles with different sizes, and the remaining striated region was the uncorroded substrate metal. After 30 days of exposure as reflected in Figure 2(c), the corrosion area continued to increase, and the surface topography was uneven. The corrosion products were dominated by spherical and irregular particles. When the Q235 carbon steel specimen was exposed for 90 days, Figure 2(d) exhibits that most areas of the substrate metal were covered by the corrosion products with further expansion of the corrosion area, and the surface topography was uneven. A number of vesicular structures in different sizes could be observed; part of them were damaged; spherical, rod-like, and needle-like corrosion products could be seen under the damaged surface. At the exposure time of 180 days, Figure 2(e) reveals that the corrosion products of the Q235 carbon steel were composed of a large number of rod-like and spherical particles; rod-like and spherical particles aggregated into clusters; clusters of the corrosion products were loose and porous; also, they were more conducive to water adsorption, which could accelerate the corrosion rate of the carbon steel. When the Q235 carbon steel sample was exposed for 365 days, Figure 2(f) demonstrates that most of the corrosion product particles had joined together to form layered corrosion products; few spherical particles remained closely connected; the surface of the rust layer appeared compact and smooth, but there were still cracks on it.

### 3.3. Microscopic Corrosion Morphology in the Section View

**Figure 3:**
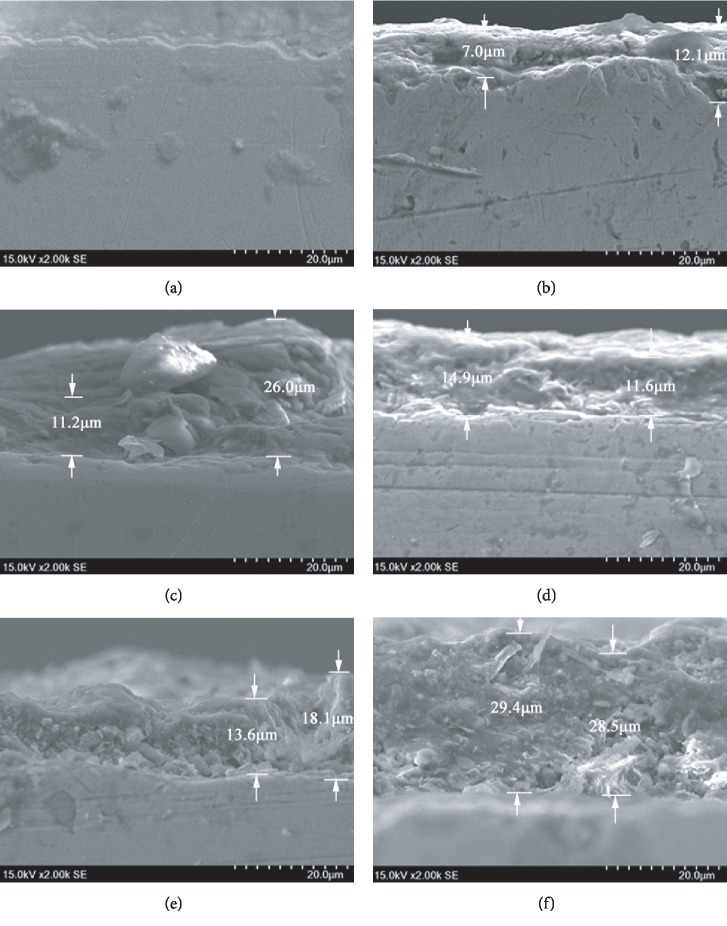
Microscopic corrosion morphologies of the Q235 carbon steel specimens in section view with different exposure time in Chengdu station: (a) 0 day, (b) 15 days, (c) 30 days, (d) 90 days, (e) 180 days, and (f) 365 days.

Figure 3 shows the microscopic corrosion morphologies of the Q235 carbon steel samples in the section view, which were exposed in Chengdu atmospheric environment for 0, 15, 30, 90, 180, and 365 days, respectively. The SEM magnifications were all set as 2000 times. For all the figures, the under part was the carbon steel substrate, and the upper part was the rust layer. As can be seen from Figure 3, the corrosion depth of the Q235 carbon steel specimen increased with the exposure time growth. As shown in Figure 3(b), at the early exposure period of 15 days, the rust layer thickness of the Q235 carbon steel specimen was small. Also, the rust layer was uneven, the largest corrosion depth reached 12.1 *μ*m, and the minimum corrosion depth was 7.0 *μ*m. When the Q235 carbon steel material was exposed for 30 days, the rust layer thickness was higher with the exposure days increasing, the corrosion thickness was between 11.2 *μ*m and 26.0 *μ*m, and the rust layer thickness was uneven, as shown in Figure 3(c). There also exist granular corrosion products of different sizes in the rust layer, which were consistent with the results of the uneven surface morphology and granular corrosion products with different sizes observed in Figure 2(c). At the exposure time of 90 days as reflected in Figure 3(d), the rust layer thickness was between 11.6 *μ*m and 14.9 *μ*m, which was smaller than that of 30 days. Also, the fluctuation of the rust layer thickness gradually became smaller. This is because most parts of the substrate metal were covered by the corrosion products, and the loose corrosion products on the surface had gradually fallen off. When the Q235 carbon steel material was exposed for 180 days, Figure 3(e) shows that the thickness of the rust layer was further increased, with a range of 13.6~18.1 *μ*m; also, the corrosion products of spherical and rod-like particles could be observed, which was consistent with the observation of the surface morphology of the rust layer in Figure 2(e).  Figure 3(f) exhibits the cross-section morphology of the Q235 carbon steel sample exposed for 365 days. As can be seen, the rust layer was thickening apparently with the depth of 28.5~29.4 *μ*m, which was fairly even. Besides, the surface of the rust layer was relatively dense, and the bottom of the rust layer was relatively loose with granular corrosion products.

### 3.4. XRD Analysis

**Figure 4:**
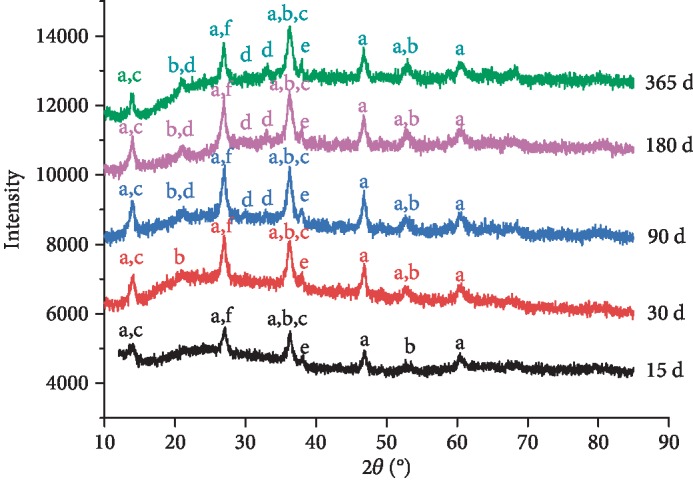
XRD spectra of the corrosion products of the Q235 carbon steel samples exposed at different periods in Chengdu station: (a) *γ*-FeOOH, (b) *α*-FeOOH, (c) Fe(OH)_3_, (d) Fe_3_O_4_, (e) *γ*-Fe_2_O_3_, and (f) FeSO_4_·*n*H_2_O.

For the samples of Q235 carbon steel exposed after different stages in Chengdu station, the surface rust residue was scraped for XRD analysis; the XRD results can be seen in Figure 4. When the Q235 carbon steel material was exposed in the atmospheric environment of Chengdu city for 15 days, the corrosion products mainly contained *γ*-FeOOH, *α*-FeOOH, Fe(OH)_3_, *γ*-Fe_2_O_3_, and FeSO_4_·*n*H_2_O. At this time, the diffraction peak of *α*-FeOOH was not obvious, which indicated that there was little corrosion product of *α*-FeOOH. For the exposure time of 30 days, the corrosion products were the same as those of 15 days; the peak intensities of all the products increased, demonstrating that the content of all the corrosion products increased. In particular, the increasing strengths of the peaks corresponding to *γ*-FeOOH and *α*-FeOOH were more obvious, showing that the contents of the corrosion products *γ*-FeOOH and *α*-FeOOH increased considerably. For the corrosion products of 90 days of exposure time and 180 days of exposure time, Fe_3_O_4_ was found in the corrosion products; the peak intensities of *α*-FeOOH and *γ*-Fe_2_O_3_ were also enhanced. When the Q235 carbon steel specimen was exposed for 365 days, the corrosion products mainly contained *γ*-FeOOH, *α*-FeOOH, Fe(OH)_3_, *γ*-Fe_2_O_3_, Fe_3_O_4_, and FeSO_4_·*n*H_2_O. Compared with the previous corrosion periods, the peak strength of the *γ*-FeOOH decreased, indicating that the content of the unstable corrosion product *γ*-FeOOH was reduced. However, the peak strengths of *α*-FeOOH, *γ*-Fe_2_O_3_, and Fe_3_O_4_ increased significantly, showing that the contents of the stable corrosion products *α*-FeOOH, *γ*-Fe_2_O_3_, and Fe_3_O_4_ became more.

Through analysis on the mechanism of chemical reaction in the process of atmospheric corrosion of carbon steel, at the initial stage of exposure, the corrosion reaction process of carbon steel was as follows:

(1) $$\begin{array}{c} \text{Anode process}:\text{Fe}⟶{\text{Fe}}^{2+}+2{\text{e}}^{‐} \end{array}$$

(2) $$\begin{array}{c} \text{Cathode process}:{\text{O}}_{2}+2{\text{H}}_{2}\text{O}+4{\text{e}}^{-}⟶4\text{O}{\text{H}}^{-} \end{array}$$

OH^−^ combined with Fe^2+^ to form Fe(OH)_2_:

(3) $$\begin{array}{c} {\text{Fe}}^{2+}+2{\text{OH}}^{-}⟶\text{Fe}{\left(\text{OH}\right)}_{2} \end{array}$$

Fe(OH)_2_ was not stable and was gradually oxidized to FeOOH by O_2_ dissolved in the electrolyte solution:

(4) $$\begin{array}{c} 4\text{Fe}{\left(\text{OH}\right)}_{2}+{\text{O}}_{2}⟶4\text{FeOOH}+2{\text{H}}_{2}\text{O} \end{array}$$

When the complete rust layer was formed on the surface of carbon steel, the corrosion reaction process was as follows:

(5) $$\begin{array}{c} \text{Anode process}:\text{Fe}⟶{\text{Fe}}^{2+}+2{\text{e}}^{‐} \end{array}$$

(6) $$\begin{array}{c} \text{Cathode process}:{\text{Fe}}^{2+}+8\text{FeOOH}+2{\text{e}}^{-}⟶3\text{F}{\text{e}}_{3}{\text{O}}_{4}+4{\text{H}}_{2}\text{O} \end{array}$$

Among the corrosion products through XRD analysis, *γ*-FeOOH was a kind of volatile corrosion product; also, it was the main component of the rust layer in the early corrosion stage of carbon steel material. It could dehydrate to form *γ*-Fe_2_O_3_. However, after long-term exposure, it could transform into Fe_3_O_4_ with more thermodynamic stability. Fe_3_O_4_ could be oxidized to *α*-FeOOH under the function of oxygen, *α*-FeOOH was a relatively stable corrosion product, and its grain was finer than that of *γ*-FeOOH. With the content of *α*-FeOOH increasing, the stability of the rust layer was enhanced, so the rust layer with longer exposure time was denser. The colloidal hydroxide Fe(OH)_3_ contained a certain amount of crystal water; it could be dehydrated to form FeOOH or Fe_2_O_3_ under certain conditions. The formation of FeSO_4_·*n*H_2_O came from the adsorption of SO_2_ in the atmosphere; it deposited on the surface of the wet rust layer and then reacted with Fe.

### 3.5. EDS Analysis

EDS surface scanning and element analysis were performed on the Q235 carbon steel samples after being exposed in Chengdu atmospheric environment for 15, 30, 90, 180, and 365 days, respectively. The EDS results are shown in Table 3; the rust layers with different exposure time had the main constituent elements of Fe and O. So the principal corrosion products of Q235 carbon steel material exposed in Chengdu station were the compounds containing Fe and O, which was consistent with the XRD analysis results of the corrosion products: *γ*-FeOOH, *α*-FeOOH, Fe_3_O_4_, and Fe_2_O_3_. At the same time, the rust layer also contained trace S, Cl, and N elements. The S element might be derived from SO_2_ or sulfate deposited in the atmospheric environment, N elements might come from NO or NO_2_ pollution, and Cl elements might come from chlorine ion pollution in the atmospheric environment.

### 3.6. FTIR Analysis

**Figure 5:**
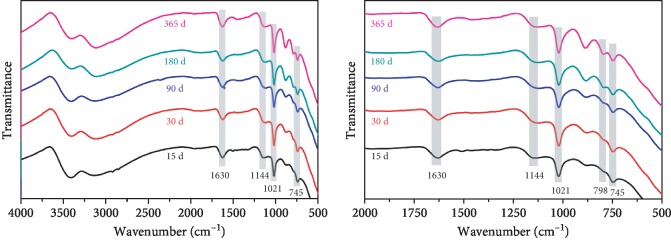
FTIR spectra of the corrosion products of the Q235 carbon steel samples exposed at different periods in Chengdu station.

FTIR spectra of the corrosion products of the Q235 carbon steel samples exposed at different periods in Chengdu station are shown in Figure 5. From the infrared spectra of Figure 5, it could be seen that the characteristics peaks at 1144 cm^−1^, 1021 cm^−1^, and 745 cm^−1^ corresponded to the characteristic absorption of *γ*-FeOOH, illustrating that one kind of the corrosion products was *γ*-FeOOH. There was a strong absorption peak in the absorption band near 1630 cm^−1^, because it was the curved vibration characteristic peak of -OH, indicating that there was a large amount of crystal water in the corrosion products. In addition, it can be seen that the characteristic absorption peak of the *α*-FeOOH at 798 cm^−1^ was gradually reflected with the exposure time increasing, indicating that in the later exposure time of 180 days and 365 days, *γ*-FeOOH started to transform to *α*-FeOOH gradually.

Among various hydroxyl oxides of iron, the electrochemical stability of *α*-FeOOH was the best, while other corrosion products such as *β*-FeOOH, *γ*-FeOOH, *δ*-FeOOH, and amorphous phase all had electrochemical activity, which were easy to be reduced. Hence, it had definite protective effect on the rust layer after the occurrence of *α*-FeOOH in the rust layer. When the Q235 carbon steel samples were exposed in high-humid atmospheric environment of Chengdu for 365 days, plenty of *γ*-FeOOH were formed. However, there was not a large number of corrosion product *α*-FeOOH detected in the rust layer because the corrosion time is not long enough. The results indicated that although the Q235 carbon steel specimen had formed a dense rust layer on the surface, its rust layer still showed the existence of electrochemical instability. Comprehensive analysis of the results of XRD and FTIR revealed that the corrosion products of Q235 carbon steel material exposed in Chengdu atmospheric environment for 365 days mainly contained *γ*-FeOOH, Fe(OH)_3_, Fe_3_O_4_, and a small amount of *α*-FeOOH; also, a lot of water of crystallization exists in the corrosion products. Furthermore, with the growth of the exposure time, *γ*-FeOOH began to transform to *α*-FeOOH, so the electrochemical stability of the rust layer tended to increase.

## 4. Conclusions

1. In the corrosion process of Q235 carbon steel material, the degree of corrosion on the front side was much greater than that on the back side. With exposure time increasing, the area of the rust layer expanded and the rust layer gradually became thicker
2. With exposure time increasing, the surface microscopic corrosion morphologies changed from irregular particles and vesicle structures to rod-shaped and spherical particles and loose clusters. Finally, the corrosion products connected to the layered structure, and the surface rust layer was obviously dense and flat
3. The corrosion products were mainly composed of *γ*-FeOOH, *α*-FeOOH, Fe(OH)_3_, *γ*-Fe_2_O_3_, Fe_3_O_4_, and FeSO_4_·*n*H_2_O. In the later exposure periods, the proportions of *α*-FeOOH, *γ*-Fe_2_O_3_, and Fe_3_O_4_ were increased

## Figures and Tables

**Table 1:** Chemical composition of Q235 carbon steel.

Chemical composition (wt%)						
Element	C	Si	Mn	S	P	Fe
wt%	0.16	0.20	0.61	0.023	0.0019	Balance

**Table 2:** The environmental parameters during the atmospheric exposure test in Chengdu.

Place	Temperature (°C)	Relative humidity (%)	Rainfall precipitation (mm/a)	pH value of rainfall	SO_2_ concentration (*μ*g/m^3^)	Cl^−^ settling rate (mg/m^2^/d)
Chengdu city	16.6	81	966.9	6.4	11.3	0.37

**Table 3:** EDS component analysis of the element weight percentage (wt%) with different exposure time.

Element (wt%)	15 days	30 days	90 days	180 days	365 days
C	10.01	12.08	10.59	6.43	11.67
N	5.16	5.44	5.57	4.66	5.06
O	37.37	35.1	35	34.96	36.2
S	0.23	0.05	0.12	0.07	0.15
Cl	0.07	0	0.1	0.07	0.1
Fe	47.16	47.33	48.62	53.8	46.83

## Data Availability

The data used to support the findings of this study are included within the article.
